# A Medical Science Educator’s Guide to Selecting a Research Paradigm: Building a Basis for Better Research

**DOI:** 10.1007/s40670-019-00898-9

**Published:** 2019-12-27

**Authors:** Megan E.L. Brown, Angelique N. Dueñas

**Affiliations:** Health Professions Education Unit, Hull York Medical School, John Hughlings Jackson Building, University Road, Heslington, York, YO10 5DD UK

**Keywords:** Paradigm, Research approach, Medical education, Methodology

## Abstract

A research paradigm, or set of common beliefs about research, should be a key facet of any research project. However, despite its importance, there is a paucity of general understanding in the medical sciences education community regarding what a research paradigm consists of and how to best construct one. With the move within medical sciences education towards greater methodological rigor, it is now more important than ever for all educators to understand simply how to better approach their research via paradigms. In this monograph, a simplified approach to selecting an appropriate research paradigm is outlined. Suggestions are based on broad literature, medical education sources, and the author’s own experiences in solidifying and communicating their research paradigms. By assisting in detailing the philosophical underpinnings of individuals research approaches, this guide aims to help all researchers improve the rigor of their projects and improve upon overall understanding in research communication.

## Introduction

There has been a recent movement within medical education towards greater methodological rigor [1, 2]. Many scholars argue that in order to achieve “academic legitimacy” [3] strong theoretical frameworks [4, 5] engaging in discussion concerning the nature of knowledge within a piece of work are required [6]. Put simply, clear research principles assist others in understanding your research.

The nature of knowledge within a piece of work is detailed and explored within a research project’s *paradigm*. A research paradigm may be defined as “the set of common beliefs and agreements shared between scientists about how problems should be understood and addressed” [7]. A paradigm is an assumption about how things work, sometimes illustrated as a “worldview” involving “shared understandings of reality” [8, 9]. Detailing one’s research paradigm is essential, as paradigms “guide how problems are solved” [10], and directly influence an author’s choice of methods. All researchers make assumptions about the state of the world before undertaking research. Regardless of whether that research is quantitative or qualitative, these assumptions are important as they impact upon the interpretation of a study’s results. Mitroff and Bonoma summarize this position and put forth “the power of an experiment is only as strong as the clarity of the basic assumptions which underlie it. Such assumptions not only underlie laboratory experimentation but social… research as well” [11]. Paradigms also assist in setting ground rules for the application of theory when observing phenomena. Such ground rules “set the scene” for research, providing information as to how best evaluate new concepts [7].

Medicine and, as a consequence, health professions education, has traditionally been conducted from a positivist or post-positivist paradigm, detailed later in this paper, both of which maintain a universal truth exists, as, “in medicine, the emphasis on… body parts, conditions and treatments assumes that these are universally constant replicable facts” [12]. Given the dominance of this belief, there has been a relative dearth of literature within medical sciences education explicitly detailing paradigmatic assumptions. This is changing, with an increasingly widespread recognition of the important role assumptions play in result interpretation and in setting ground rules, both in research and in classrooms [13, 14]. As such, explicitly acknowledging one’s paradigm is becoming an expected element of medical science education research.

**Fig. 1:**
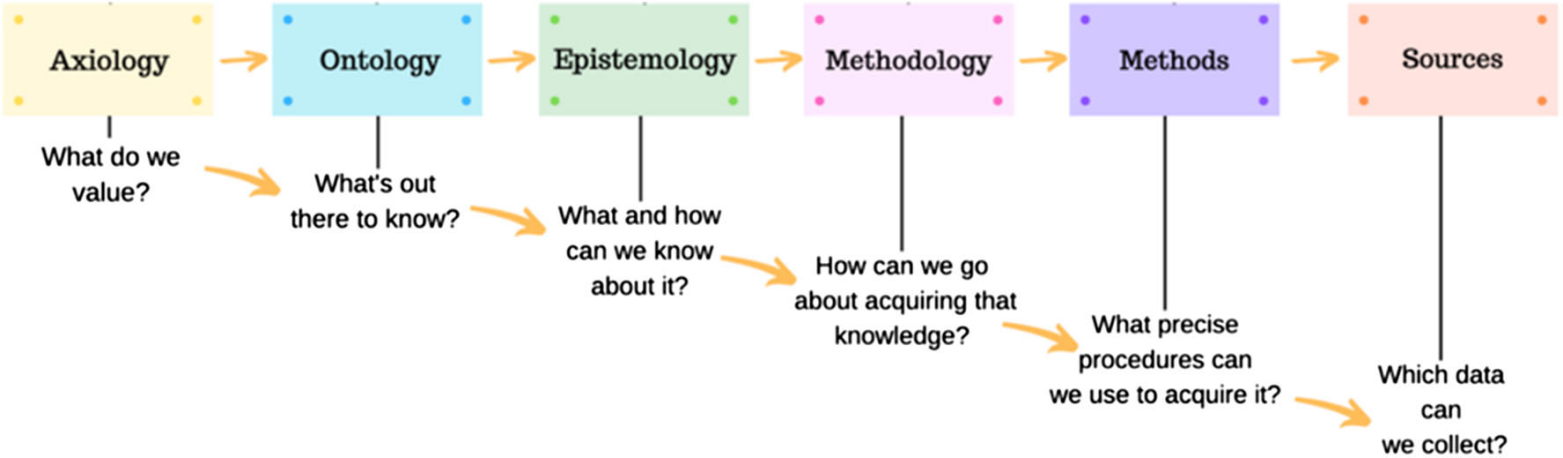
The building blocks forming a piece of work’s research paradigm and how they interrelate. Image is an adapted version of Grix’s paradigmatic building blocks [15]. Image adapted by authors to include axiology as an important block not originally detailed

In order to detail your work’s paradigm, it is important to consider what a paradigm consists of. The paradigm of a piece of work is constructed of several “building blocks,” detailed in Fig. 1. The first set of these building blocks (axiology, ontology, epistemology, methodology) are composed of philosophical assumptions that “direct thinking and action” such as selecting one’s methods [16].

Axiology, the first “brick” in the construction of a project’s paradigm involves the study of value and ethics [17]. Once an area of value to study has been identified, and research ethics considered, ontology, which questions “the nature of reality” [3] must be contemplated. Once you possess a firm philosophical understanding of your study area’s reality, the nature of knowledge within that reality needs determining—this is known as the epistemology of a piece of work.

Frank discussion of a work’s ontology and epistemology allows an appropriate methodological approach to be selected and reduces the ambiguity surrounding result interpretation [18]. Without such regulation “even carefully collected results can be misleading” as the “underlying context of assumptions” is unclear [19]. This monograph will detail a series of considerations, forming a how-to guide, for selecting an appropriate paradigm for your medical sciences education research.

## Select your Research Paradigm Before You Begin Researching

Given that paradigms inform the design of, and fundamentally underpin, both quantitative and qualitative research, it is important to select your paradigm before you begin researching. Teherani et al. emphasize the need for this nicely: “alignment between the belief system underpinning the research approach, the research question, and the research approach itself is a prerequisite for rigorous… research” [20]. Such alignment can only be assured prospectively.

One frequently cited argument for not considering the research paradigm of a piece of work is the time-consuming nature of this process. Admittedly, selecting a research paradigm does (and should if done well) take time. Ensure you factor this consideration into your plans when drafting a timeline for your research project. It is difficult to provide guidance on how much time one should spend selecting a research paradigm as, depending upon the project in question and research team, this may vary. We recommend threading consideration of your research paradigm into the “design” phase of your research. Using the present work will also contribute to reducing the time-consuming aspect of this work; for many novices, approaching the language and process of paradigms can prove daunting and take time. However, this work is designed to ease that process.

## Try Thinking About Research Paradigms Using the Metaphor of a Glass Box

Research paradigms can seem overwhelming—indeed, even experienced academics may struggle to distinguish between the various building blocks constituting a paradigm. Thinking of one’s research paradigm using the metaphor of a glass box, as described by Varpio [21], may assist in better visualizing and understanding the constituent elements of a paradigm. Using this metaphor, your paradigm is the glass box in which you stand, framing how you see the outside world. One’s beliefs regarding the ontology and epistemology of knowledge color the glass box in different ways, lending different lights to the same situation for different individuals. Given this, you may research a topic using a different approach to your colleague within the same area.

## Think About your Reason for Carrying Out the Research

This may seem like an obvious consideration, but it is an area that is often not consciously reflected upon within medical science education research. What is your motivation to study this topic? Have you been practically, academically, or politically motivated? In other words, is it something you have noticed in your day to day work that requires further study; are you simply passionate to know more; or is there a political “hot topic” you or others are interested in researching?

Building upon your initial thoughts regarding your motivation, try to reflect more deeply regarding what you are really trying to achieve. Chilisa compares different paradigmatic reasons for doing research, as can be seen in Table 1 [23]. Thinking of your own reason for doing research and comparing this with Chilisa’s reasons should begin to cast light on which paradigm may be an appropriate choice for your research.

**Table 1:** Adapted from Chilisa’s comparison of paradigmatic reasons for doing research [22]

Paradigm	Positivist and post-positivist	Constructivist	Critical theory
Reason for doing the research	To discover laws that are generalizable and govern the universe	To understand and describe human nature	To destroy myths and empower people to change society radically

## Consider your Axiological Approach

The next step in the consideration of an appropriate paradigm for your research is reflecting upon your axiological approach. Traditionally, Guba and Lincoln describe a paradigm as involving three building blocks: ontology, epistemology, and methodology [24]. However, there has been a move towards including axiology as a fourth defining characteristic of a paradigm [25]. Axiology involves ethical considerations and “asks what ought to be” within a field of research [26]. It is an important starting point for any proposed research, as it considers what would be of value to research and how to go about conducting ethical research within that area [27]. Given this, we modified Grix’s paradigmatic building blocks [15] to include axiology as a key early consideration in paradigm selection (Fig. 1).

Considering your axiological approach is best done in a designated reflective space with all members of your research team during the planning phase of a research proposal. Building on considering your purpose in doing research, you must consider the personal values informing your proposal. Ask yourself the following:

- Why is this research worth my time and attention?
- What motivates me? Am I driven by imperatives (e.g. funding, social justice)?
- Or, do I believe education to be inherently valuable, providing justification for any research that informs educational practice? [28]

Once the values underpinning your inquiry are clear and it is evident your research is justified, potential ethical issues should also be considered. For example, if your axiological reflection reveals you are being driven by an external motivator, it may be appropriate to disclose this within your research design. Most journals mandate inclusion of detail regarding any funding underpinning your research and any conflicts of interest (which could include sources of personal funding). Kirkman et al. include a detailed “competing interests” statement in their systematic review evaluating the outcomes of recent patient safety interventions for junior doctors and medical students [29]. Particularly relevant are two author’s affiliations with the General Medical Council (GMC), the UK’s regulatory body for physicians, and consultancy work several authors had undertaken previously on the topic of patient safety for a variety of institutions. These institutional affiliations could color the author’s perspectives and interpretations in tacit ways, in line with institutional values. As such, considering any such competing interests or associations within your team’s axiological reflection is the key.

## Reflect upon your Ontological Assumptions

We all hold ontological assumptions, even if we do not explicitly consider or detail them. Reflecting upon them allows you to choose a paradigm in keeping with your beliefs regarding the nature of reality [3]. Reality refers to the social world in which you wish to conduct your research [22].

Different paradigms adopt different approaches to defining the nature of reality. There are many paradigms research may operate within, with some scholars even attempting to define new, albeit contested, paradigms within the social sciences in recent years [30]. Given this, detailing the ontology of every available paradigm is beyond the scope of this article. Instead, we will focus upon the four paradigms most commonly used within general medical education [3]: positivism, post-positivism, constructivism/interpretivism, and critical theory.

To assess your ontological assumptions, ask yourself this: do you believe there is “one verifiable reality,” or that “multiple socially constructed realities” exist? [21, 31] The former stance is sometimes referred to as a “realist” ontological position, with the latter stance known as “anti-realism” or “relativism” [32]. Broadly speaking, the four paradigms most commonly used within medical education fall into either of these two categories, but there are differences in how they frame their position, detailed in Table 2.

**Table 2:** Ontological assumptions of positivism, post-positivism, constructivism/interpretivism, and critical theory [30, 33–39]

Paradigm	Positivist	Post-positivist	Constructivist/interpretivist	Critical theory
Ontological assumptions	There is a single, objective reality that can be observed through science.	There is a single, objective reality. However, scientific observations involve error so reality can only be known imperfectly.	There are multiple subjective realities, each of which is socially constructed by and between individuals.	There are multiple subjective realities influenced by power relations in society. Reality is shaped by social, political, cultural, economic, ethnic, and gender values.

## Reflect upon your Epistemological Assumptions

Once you are aware of your assumptions regarding the nature of reality, reflecting upon your epistemological assumptions regarding the nature of knowledge is necessary. When considering your research epistemology, it may be useful to reflect upon “what counts as knowledge within the world” [40]. Epistemology seeks to answer two questions—one, *what is knowledge*, and two, *how is knowledge acquired*? [41].

Again, the epistemological approaches of positivism, post-positivism, constructivism, and critical theory differ. These are outlined within Table 3.

**Table 3:** Epistemological assumptions of positivism, post-positivism, constructivism/interpretivism, and critical theory [27, 34]

Paradigm	Positivist	Post-positivist	Constructivist/interpretivist	Critical theory
Epistemological assumptions	Neutral knowledge can be obtained through the use of reliable and valid measurement tools.	Obtaining knowledge is subject to human error. Therefore, human knowledge is imperfect and only “probable” truths can be established.	Knowledge is subjective and formed at an individual level.	Knowledge is also subjective, but created and negotiated between individuals and within groups.

## Become Familiar with Different Types of Paradigm to Evaluate Where You and Your Work Fit

Above, we have focused on positivism, post-positivism, constructivism, and critical theory as four common paradigms in medical education [37]. These are only a subset of paradigms that might align with an individual’s medical education research aims [42]. We recommend researchers to familiarize themselves with as many different types of paradigms as possible, to best understand where you as a researcher, but also your team and project fit.

Given the complexity of paradigms, rather than delving too deeply into the nuances of philosophy associated with paradigms, seeking simple infographics and metaphors can make exploration more manageable. We have already introduced some simple tables and the glass house metaphor [21], but you may find it helpful to seek other visualizations, such as the

“research onion” [43, 44]. In brief, the “research onion” depicts paradigmatic considerations as layers, in lieu of building blocks or glass walls.

Another helpful way to explore paradigms is to be mindful of such in your own reviews of literature. Are authors explicitly discussing their paradigms? If so, do you agree? If not, how would you categorize their paradigm based on their study details? Zaidi and Larsen provide an excellent commentary where they categorize papers based on research paradigms, using their own interpretations [45]. Such an activity may prove useful to those wishing to improve their understanding of paradigms, in a practical fashion.

## Use your Chosen Paradigm to Select an Appropriate Methodology

How you can go about “acquiring” knowledge, so that it aligns naturally with your paradigm, might be considered next. For example, if an individual is a strict positivist, believing that there are single truths, and that such truths can be measured, you would expect them to utilize stricter forms of experimental research, with explicit hypothesis testing. Different methodologies align best with different paradigms [46].

Consideration of research teams’ methodologies can also be helpful in understanding your paradigm, prior to moving forward with research projects. Following the example above, if your research team most often utilizes experimental design in your projects, what might this say about your regard for what knowledge and information you place value in?

## Examine your Methodology in Order to Select an Appropriate Data Gathering Technique

Too often, methodology and methods are used interchangeably by novice researchers, when they should be regarded as distinct concepts [47]. Methodology is the strategy or overall plan to acquire knowledge, and methods are the actual techniques used to gather and analyze data [33].

For example, a research team interested in examining interprofessionalism in a healthcare setting may identify most with a constructivist paradigm, believing reality is subjectively constructed by individuals. Such a team might consider ethnography to be an appropriate methodology. But the actual research methods they undertake might be a variety of observations with field notes, audio or video recordings, or qualitative interviews [48]. These methods align with the methodology, although eventual selection of methods may also be highly associated with the practicality of such techniques, in addition to paradigm considerations.

**Fig. 2:**
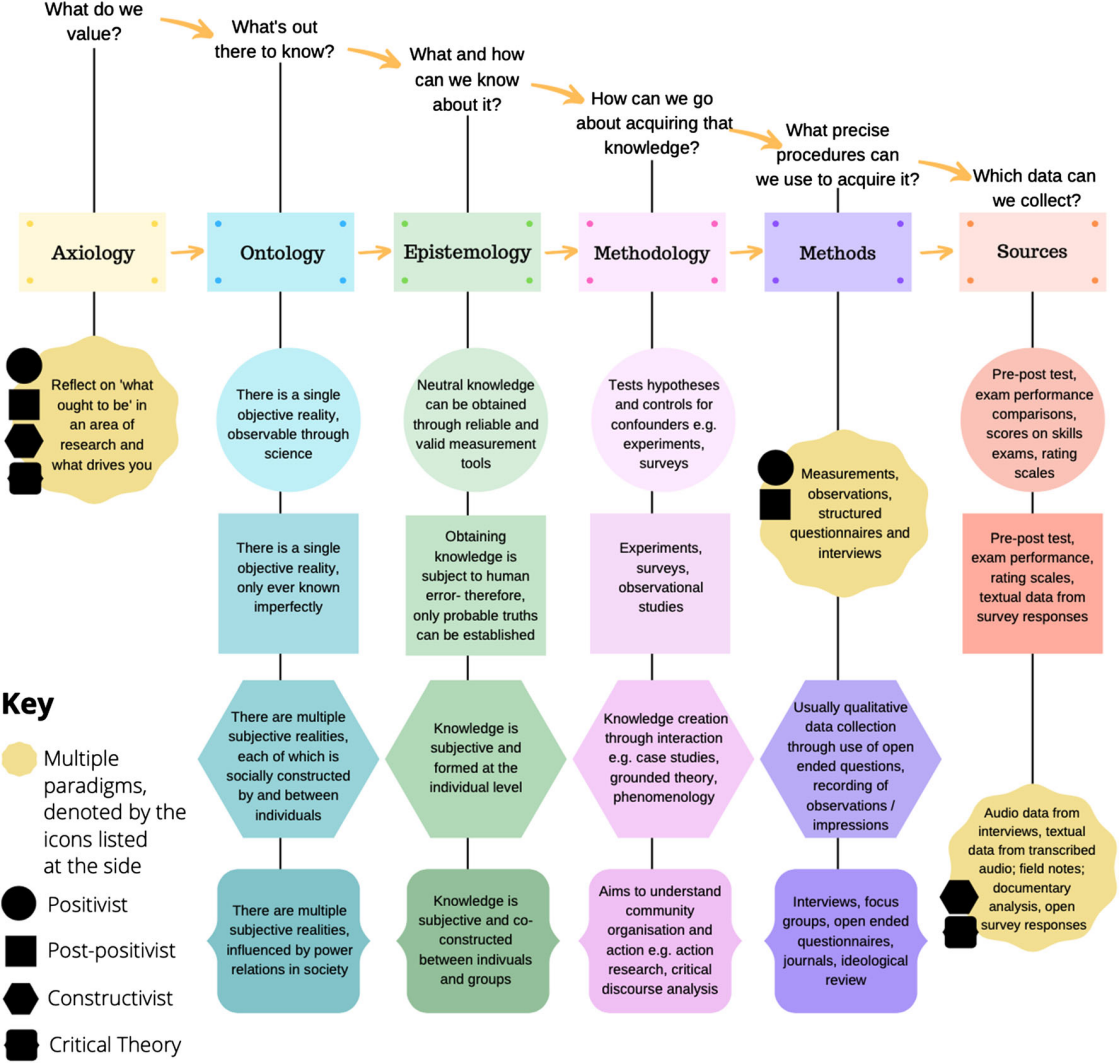
The building blocks of a research project’s paradigm within the four main medical science education paradigms summarized. Each shape in the figure refers to one of the four main medical science paradigms. Each color refers to an element of a piece of research’s paradigm. Please see the key to this figure to aid with interpretation

The above sections have provided an overview of the “building blocks” of a research project’s paradigm. For ease of reference, these building blocks are summarized for the four main paradigms used within medical science education, in Fig. 2 [30, 36, 49, 50].

## Clearly Detail Your Paradigm and its Building Blocks When You Write about your Research

A paradigm does no good if it only exists in the mind of the researcher and is not clearly communicated. Clearly detail your paradigm, for your own understanding as a researcher. It is often helpful to describe your paradigm by answering the questions outlined in the building blocks, as shown in Fig. 1.

But also keep in mind to make any details of your paradigm accessible and understandable for your target audience when disseminating your research. Depending on the scope and goals of your research, description of your paradigm could range from a paragraph or two in a research report designed for publication, to a multipage subchapter of a larger report or thesis assignment. In either case, writing about the paradigm is key for the audience to understand the context of your research, although the level of detail in which you communicate your paradigm may vary.

Locating accessible literature to draw upon when writing about your paradigm can prove difficult. The field is littered with philosophical jargon that can act as a barrier to entry into the world of paradigms, as earlier addressed in time consideration of paradigm selection. We hope this guide will assist you in beginning to understand some of the foundational terms within this field. If you are interested and have time, there is a wealth of literature within the field of “Philosophy of Science” that explicitly discusses the nature of knowledge and varying paradigmatic stances. Some seminal texts include *The Foundations of Social Research* [36], *The Structure of Scientific Revolutions* [7], *Bruno Latour: Hybrid thoughts in a Hybrid world* [51], and *The Paradigm Dialog* [52].

Several introductory textbooks and articles offer integrated summaries of these seminal texts including, but not limited to Kivunja and Kuyini’s *“Understanding and Applying Research Paradigms in Educational Contexts”* [53]; Avramidis and Smith’s “An introduction to the major research paradigms and their methodological implications for special needs research” [54]; Denzin and Lincoln’s *The Sage Handbook of Qualitative Research* [55]; and *Philosophy of Science: A Very Short Introduction* [56].

## Move from Philosophy to Practicality

For those involved in the day-to-day aspects of healthcare teaching, many times one of the first questions that comes to mind around the philosophical underpinnings of research is: how can this be practically applied to my work? Beyond improving rigor and understanding, as thoroughly discussed, there are two key ways to approach the practical side of research: from the before and the after.

**Fig. 3:**
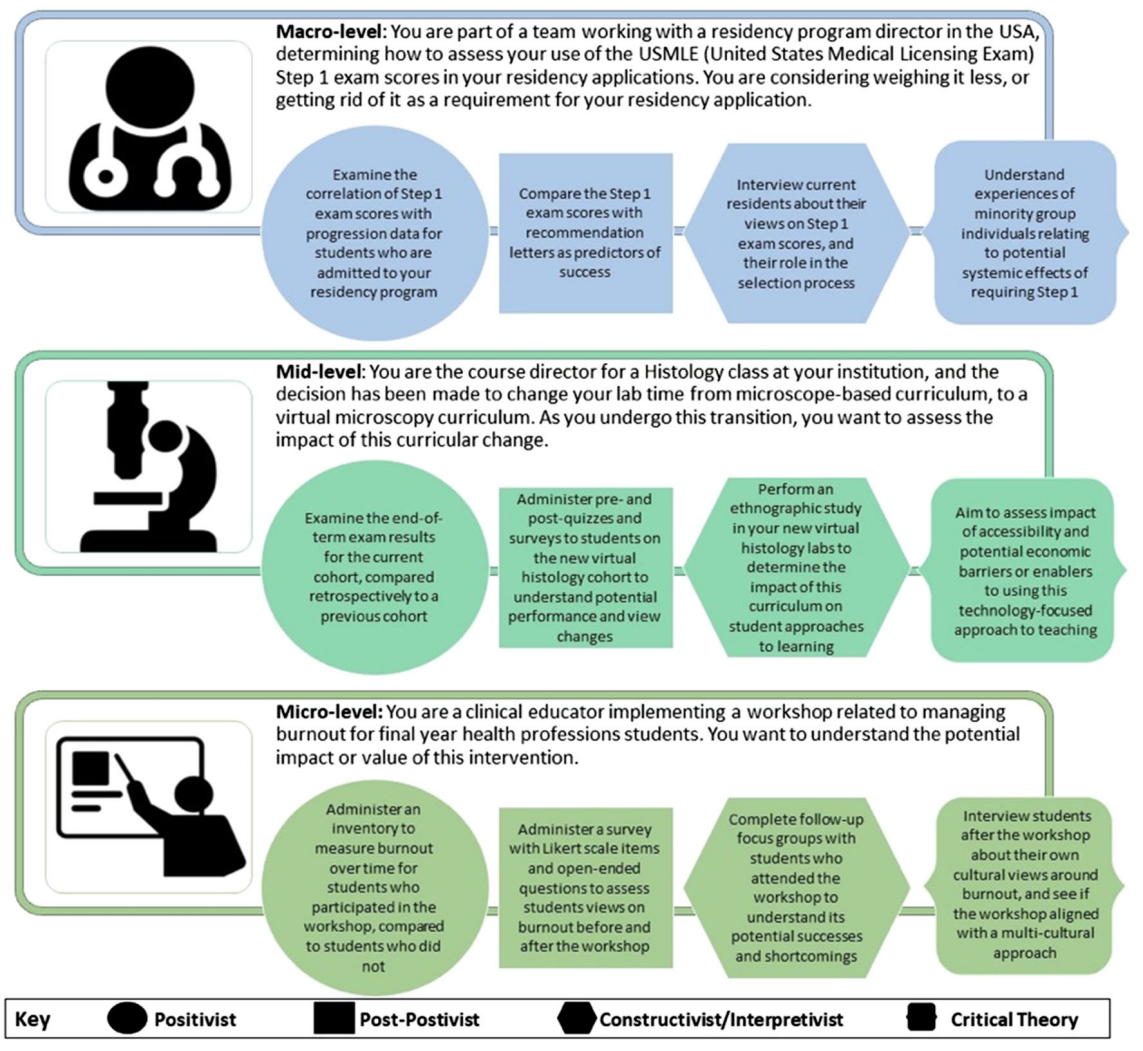
Examples of real-world educational scenarios at a macro-, mid-, and microlevel and how consideration of different paradigms could be aligned to varying research aims and processes

Considering the practical problems and questions you face as a medical sciences educator, then considering how different paradigms could be used to approach problems in different ways, is a practical “before” way to consider paradigms. To elucidate the ways in which real-world problems can be approached from a paradigm-informed perspective, we’ve included some examples in Fig. 3. For somevarious real-world examples, at different educational levels, we have provided some different examples of research approaches, that would naturally align with different paradigms.

From the “after” research perspective, praxeology is the last -ology you may wish to reflect upon. Concerned with the more practical recommendations that often arise *from* research, praxeology is concerned with not just understanding human actions, but interpreting them in meaningful ways [45]. If your research has contributed to “knowledge,” what does this mean for your day-to-day role as a medical sciences educator? In this way, practicality can be also important after the research process. Using the mid-level example from Fig. 2, if you completed research from a constructivist approach, you may have discovered that self-guided methods in virtual histology labs was not leading to a conducive learning environment. This may lead to your decision to create video guides to accompany virtual histology resources, so students have instructor-led examples to initially guide their learning.

In addition to the above ways of practically approaching paradigms, researchers may also wish to contemplate the practical paradigm of pragmatism. Pragmatism focuses on research outcomes and, as such, does not place value on considering either epistemology or ontology. Instead, pragmatism strives to focus on what works best for understanding and solving problems [57]. Pragmatists rely on the methods that work best in practice to answer specific research questions, focusing most heavily on the practicalities of the chosen approach, not just paradigmatic alignment [58]. However, it is the view of some that pragmatism should be viewed as more of an approach, rather than a “true” paradigm. Consequently, the present work has not explored pragmatism in detail as it has other common paradigms [30].

## Collaborate with or Consult Experienced Researchers Where Possible

While paradigms might seem complex and novel for many in the medical education community, they are a key facet of research, and certainly not new to other disciplines, such as sociology and general education [59–61]. Given this, collaboration can prove fruitful and may be the final key to success. When possible, collaborating with experienced researchers, particularly those who focus upon methodology, can be very beneficial. Experienced scholars can provide guidance regarding the philosophical questions associated with paradigms, while keeping in mind which methodology and methods may be best utilized by the research team. Where collaboration is not feasible, you may wish to contact a methodologist or experienced researcher to enquire as to whether they provide consultation services to review your research approach.

Although immensely helpful for those wishing to develop their research skills, collaboration with regard to paradigm choice can generate tension, especially if researchers disagree concerning which paradigm would be best suited for their research. We recommend that, prior to agreeing upon any collaborative projects, potential collaborators meet to develop a “shared agenda.” Shared agendas include a set of common objectives, a list of available resources, research questions of interest, and discussion as to each researcher’s personal paradigm. Compromise may be required on the behalf of one, or several, researchers, who may need to research within a paradigm unfamiliar to their personal stance, but best befitting the shared agenda of the collaborative team. For example, if you consider yourself to be a strict pragmatist, as introduced above, you might find extensive discussions about ontology and reality to be an unproductive use of research time. However, if working with a team of interpretivists, this may be viewed as a key part of their research efforts and study design. Through recognizing personal stances and being able to clearly express them in a dedicated reflexive space, collaboration may be eased, and even enhanced.

Lastly, when writing for publication, we recommend transparency as to each team member’s paradigmatic stance and inclusion of detail regarding how reflexivity was used to navigate any tensions. This monograph may be used as an example of collaborative writing. The authors approached this topic neutrally but have different personal paradigms. One author (MB) is a constructivist, and the other (AD) is a pragmatist. In the conception and construction of this work, the authors began with reflexive discussions on their paradigmatic assumptions, including personal views regarding the philosophy of science discussed in this paper. It was determined the shared agenda of this work was to remain as neutral as possible, while acknowledging potential assumptions each author holds. We hope this allows for a more transparent presentation of this monograph.

## Conclusions

While initially complex, identification of a research paradigm is an essential aspect of any rigorous research project. Further, beyond individual projects, association of knowledge with specific paradigms may lead to a better overall understanding of research within medical education, furthering the advancement of the entire field.

Through this article, we have attempted to outline some initial tips for researchers looking to improve on projects via identification of a research paradigm. With consideration of these tips, and more open discussions within research teams, your research can take on new purpose and be understood with greater depth.

## References

[1] Varpio L, Ajjawi R, Monrouxe LV, O'Brien BC, Rees CE (2017). Shedding the cobra effect: problematising thematic emergence, triangulation, saturation and member checking. Med Educ.

[2] Todres M, Stephenson A, Jones R (2007). Medical education research remains the poor relation. BMJ.

[3] Bunniss S, Kelly DR (2010). Research paradigms in medical education research. Med Educ.

[4] Monrouxe L, Rees C (2009). Picking up the gauntlet. Med Educ.

[5] Bordage G (2009). Conceptual frameworks to illuminate and magnify. Med Educ.

[6] Lingard L (2007). Qualitative research in the RIME community: critical reflections and future directions. Acad Med.

[7] Kuhn TS. The structure of scientific revolutions. Chicago and London 1962.

[8] Rossman GB, Rallis SF. Learning in the field: an introduction to qualitative research: Sage; 2011.

[9] Szyjka S. Understanding research paradigms: trends in science education research. Problems of Education in the 21st Century. 2012;43.

[10] Schwandt TA (2007). The sage dictonary of qualitative inquiry.

[11] Mitroff I, Bonoma TV (1978). Psychological assumptions, experimentation, and real world problems: a critique and an alternate approach to evaluation. Eval Q.

[12] Alderson P (1998). The importance of theories in health care. BMJ..

[13] Colliver JA (1996). Science in the postmodern era: postpositivism and research in medical education. Teaching and Learning in Medicine: An International Journal.

[14] Mann KV (2011). Theoretical perspectives in medical education: past experience and future possibilities. Med Educ.

[15] Grix J (2002). Introducing students to the generic terminology of social research. Politics.

[16] Mertens DM. Research and evaluation in education and psychology: integrating diversity with quantitative, qualitative, and mixed methods: Sage publications; 2014.

[17] Biedenbach T, Jacobsson M (2016). The open secret of values: the roles of values and axiology in project research. Proj Manag J.

[18] Weaver K, Olson JK (2006). Understanding paradigms used for nursing research. J Adv Nurs.

[19] Heinrich B. In a patch of fireweed: a Biologist's life in the field: Harvard University Press; 2009.

[20] Teherani A, Martimianakis T, Stenfors-Hayes T, Wadhwa A, Varpio L (2015). Choosing a qualitative research approach. J Grad Med Educ.

[21] Royal College of Physicians and Surgeons of Canada. KeyLIME (Key Literature in Medical Education) podcasts. In: Varpio L, editor. Methods consult with Lara Varpio - Episode 6. International Clinical Educators (ICE) Blog2019.

[22] Blaikie N, Priest J. Designing social research: the logic of anticipation: John Wiley & Sons; 2019.

[23] Chilisa B. Indigenous research methodologies: Sage Publications; 2011.

[24] Guba EG, Lincoln YS. Competing paradigms in qualitative research. In N. K. Denzin & Y. S. Lincoln, editors. Handbook of qualitative research. Thousand Oaks, CA: Sage; 1994. (105-117).

[25] Heron J, Reason P (1997). A participatory inquiry paradigm. Qual Inq.

[26] Deane P. A guide for interdisciplinary researchers: adding axiology alongside ontology and epistemology. Integration and Implementation Insights. 2018. https://i2insights.org/2018/05/22/axiology-and-interdisciplinarity/. Accessed 5th August 2019.

[27] Patterson ME, Williams DR (1998). Paradigms and problems: the practice of social science in natural resource management.

[28] Taber K. Classroom-based research and evidence-based practice: an introduction: Sage Publications Limited; 2013.

[29] Kirkman MA, Sevdalis N, Arora S, Baker P, Vincent C, Ahmed M (2015). The outcomes of recent patient safety education interventions for trainee physicians and medical students: a systematic review. BMJ Open.

[30] Morgan DL (2007). Paradigms lost and pragmatism regained: methodological implications of combining qualitative and quantitative methods. J Mixed Methods Res.

[31] Patton MQ. Qualitative research and evaluation methods . Thousand Oakes. ca: sage; 2002.

[32] Chalmers D, Manley D, Wasserman R. Metametaphysics: new essays on the foundations of ontology. Oxford University Press; 2009.

[33] Scotland J (2012). Exploring the philosophical underpinnings of research: relating ontology and epistemology to the methodology and methods of the scientific, interpretive, and critical research paradigms. Engl Lang Teach.

[34] Mertens DM. Transformative research and evaluation: Guilford press; 2008.

[35] Ponterotto JG (2005). Qualitative research in counseling psychology: a primer on research paradigms and philosophy of science. J Couns Psychol.

[36] Crotty M. The foundations of social research: meaning and perspective in the research process: Sage; 1998.

[37] Bergman E, de Feijter J, Frambach J, Godefrooij M, Slootweg I, Stalmeijer R (2012). AM last page: a guide to research paradigms relevant to medical education. Acad Med.

[38] Trochim WMK. Research methods knowledge base. 2nd ed. Social research methods: 2006.

[39] Bogdan RC, Biklen SK (2007). Research for education: an introduction to theories and methods.

[40] Cooksey RW, McDonald GM. Surviving and thriving in postgraduate research. Springer; 2011.

[41] Chilisa B, Kawulich B. Selecting a research approach: paradigm, methodology and methods. C Wagner, B Kawulich, & M Garner, Doing social research: a global context 2012:51–61.

[42] Frey BB. The SAGE encyclopedia of educational research, measurement, and evaluation: SAGE Publications; 2018.

[43] University of Derby. Research onion diagram. University of Derby. 2019. https://onion.derby.ac.uk/. 2019.

[44] Saunders M, Lewis P, Thornhill A. Research onion. Research methods for business students 2009:136–162.

[45] Zaidi Z, Larsen D (2018). Commentary: paradigms, axiology, and praxeology in medical education research. Acad Med.

[46] Patel S. The research paradigm–methodology, epistemology and ontology–explained in simple language. July 15th Available at: http://salmapatel co uk/academia/the-research-paradigm-methodologyepistemology-and-ontology-explained-in-simple-language (Accessed: 1/6/17). 2015.

[47] Wahyuni D (2012). The research design maze: understanding paradigms, cases, methods and methodologies. Journal of applied management accounting research.

[48] Pope C (2005). Conducting ethnography in medical settings. Med Educ.

[49] Pillay M (1997). The curriculum of practice: a conceptual framework for speech-language therapy and audiology practice with a black African first language clientele. S Afr J Commun Disord.

[50] Young S. Paradigm accommodation in water pollution assessment.

[51] Blok A, Jensen TE. Bruno Latour: hybrid thoughts in a hybrid world. Routledge; 2011.

[52] Guba EG, editor. The paradigm dialog. Alternative Paradigms Conference, Mar, 1989, Indiana U, School of Education, San Francisco, CA, US; 1990: Sage Publications, Inc.

[53] Kivunja C, Kuyini AB (2017). Understanding and applying research paradigms in educational contexts. Inte J higher educ.

[54] Avramidis E, Smith B (1999). An introduction to the major research paradigms and their methodological implications for special needs research. Emot Behav Diffic.

[55] Denzin NK, Lincoln YS. The Sage handbook of qualitative research. Sage; 2011.

[56] Okasha S. Philosophy of science: very short introduction: Oxford University Press; 2016.

[57] Creswell JW, Poth CN. Qualitative inquiry and research design: choosing among five approaches: Sage publications; 2016.

[58] Onwuegbuzie AJ, Leech NL (2005). On becoming a pragmatic researcher: the importance of combining quantitative and qualitative research methodologies. Int J Soc Res Methodol.

[59] Bergman MM (2010). On concepts and paradigms in mixed methods research. J Mixed Methods Res.

[60] Mack L. The philosophical underpinnings of educational research. Polyglossia; 2010.

[61] Taylor PC, Medina M (2011). Educational research paradigms: from positivism to pluralism. Coll Res J.

